# Association between chemotherapy and prognostic factors of survival in hepatocellular carcinoma: a SEER population-based cohort study

**DOI:** 10.1038/s41598-021-02698-x

**Published:** 2021-12-09

**Authors:** Meiqi Liu, Mengying Xu, Tiantian Tang

**Affiliations:** 1 Department and Institute of Infectious Disease, Xi’an Children’s Hospital, Xi’an Jiaotong University, Xi’an, 710003 China; 2 Department of Cardiology, Guangzhou Women and Children Medical Center, Guangzhou, China

**Keywords:** Cancer, Diseases, Risk factors

## Abstract

Hepatectomy and transplantation are the main surgical therapies for HCC patients, and radiotherapy or chemotherapy is often used as adjuvant treatment. Researches have evaluated the independent predictors of HCC, but evidence for factors predicting the efficacy of chemotherapy is rare. Patients diagnosed with HCC between 2010 and 2015 from the SEER database were included and randomly divided into non-chemotherapy and chemotherapy groups. The predictors of CSS and OS were analyzed with the Cox proportional-hazards regression model and Fine and Gray’s competing risk model. Although there was no significant difference in survival analysis between the chemotherapy and non-chemotherapy groups, the cumulative cancer-specific mortality of most HCC patients was decreased in the chemotherapy group. AJCC stage, tumor size, grade, surgery and radiotherapy were predictors of OS and CSS in the non-chemotherapy group, while AJCC stage, tumor size, AFP, grade and surgery in the chemotherapy group. Surgery combined with chemotherapy was applicable to all AJCC stage patients. Surgery was the major treatment option for patients in AJCC I and AJCC II stage, and chemotherapy in AJCC III and AJCC IV stage. In conclusion, the study provided population-based estimates of the prognostic factors in HCC patients with or without chemotherapy.

## Introduction

According to Global Cancer Statistics 2018^1^, liver cancer is the sixth most commonly diagnosed cancer and the fourth leading cause of cancer death worldwide (8% of total cancer deaths). Primary liver cancer includes hepatocellular carcinoma (HCC), intrahepatic cholangiocarcinoma (ICC) and other types. HCC comprises approximately 75–85% of all liver cancers, while ICC approximately 10–15%. The incidence of liver cancer increased in many eras of the world between 1978–1982 and 2008–2012, particularly in Oceania, North and South America and in much of Europe. While the incidence rates declined in many Asian countries^2^. Kaplan–Meier methods and the Cox proportional-hazards regression model are the most widely used method of survival analysis^3^. However, cancer-specific death and death of other causes are common in the outcomes of patients with liver cancer. These two kinds of death are competing events, one of which will prevent the occurrence of the other. Traditional survival analysis can only consider one endpoint, which may overestimate the risk of interesting events, so we used the Fine and Gray model based on the sub-distribution hazard to solve this problem at the same time^4–6^.

Currently, hepatectomy and liver transplantation are the main surgical therapies for patients with HCC. And radiotherapy or chemotherapy is often used as adjuvant or palliative treatment. The American Association for the Study of Liver Diseases (AASLD) suggested that adjuvant therapy should not be routinely used in patients with liver cancer following successful resection or ablation^7^. And several studies have analyzed the independent predictive factors of HCC by utilizing traditional survival analysis or competing risk analysis^8–11^. However, evidence for factors that could predict the efficacy of chemotherapy is still lacking.

Thus, our objective of this study is to evaluate the predictors associated with the survival of patients with HCC, who were registered within the SEER database grouped by chemotherapy through the competing risk model and the Cox proportional-hazards regression model.

## Materials and methods

### Data source

Specific clinicopathological data and prognostic outcomes of HCC patients from 2010 to 2015 were retrieved from the SEER database. This study did not require a local ethics approval or a statement. Because all the data used in this study were retrieved from the SEER database with a publicly available approach.

### Patients

**Figure 1:**
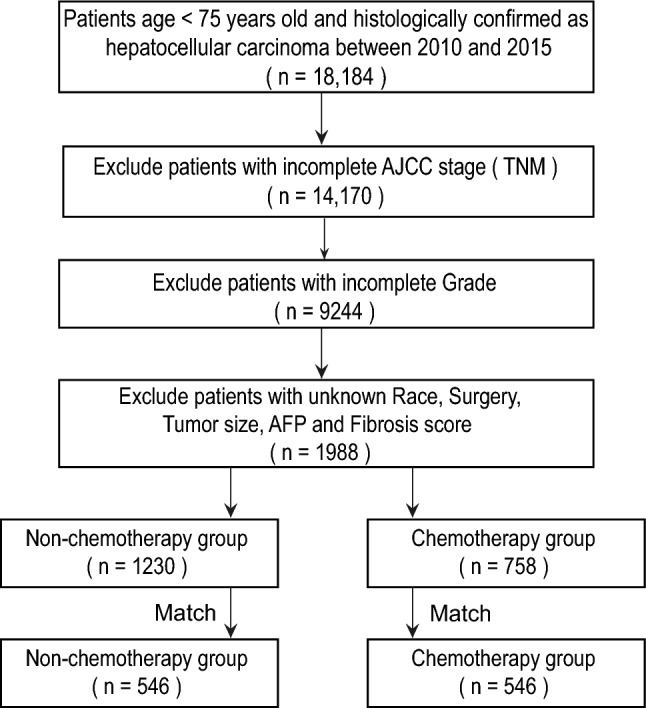
Flowchart of patients selection. AJCC, American Joint Committee on Cancer (7th). AFP, alpha-fetoprotein.

The identification of HCC was based on the International Classification of Disease for Oncology. The inclusion criteria included: (1) the patients were diagnosed between 2010 and 2015; (2) age < 75 years old; (3) primary site code C22.0; (4) histologic type code 8170-8175; (5) complete survival data. The exclusion criteria included: (1) incomplete AJCC7th tumor-node-metastasis (TNM) stage; (2) unknown SEER cause-specific death classification; (3) unknown grade and tumor size; (4) unknown AFP and fibrosis score; (5) unknown race; (6) unknown treatment. (Fig. 1).

All included patients (n = 1988) were divided into two groups according to whether they receive chemotherapy. Chi-square test was used for the comparison of these categorical variables in two groups. Race, AJCC TNM stage, grade, tumor size, AFP, fibrosis score, radiation and surgery were statistically different. So we matched the two groups of data with propensity score matching (PSM) method to eliminate these differences.

### Clinical variables of patients

Information on demographic factors (race, age and sex), tumor-related factors (tumor diameter, grade and AJCC staging system), therapeutic factors (surgery, chemotherapy, and radiotherapy) and follow-up were collected from the SEER database. Based on the Surgery Codes of the SEER program, we divided the surgical procedures into four categories: no surgery, hepatectomy, transplantation and other surgical procedures (e.g., Heat-Radio-Frequency ablation (RFA), and Percutaneous Ethanol Injection (PEI)).

OS and CSS were the interesting endpoints. The specific cause of death was based on the code of “SEER cause-specific death classification” in the SEER database. OS was calculated from the date of diagnosis to the date of death caused by any cause or the most recent follow-up. CSS was defined as the interval between the date of diagnosis and date of death due only to HCC or the most recent follow-up.

### Statistical analysis

**Figure 2:**
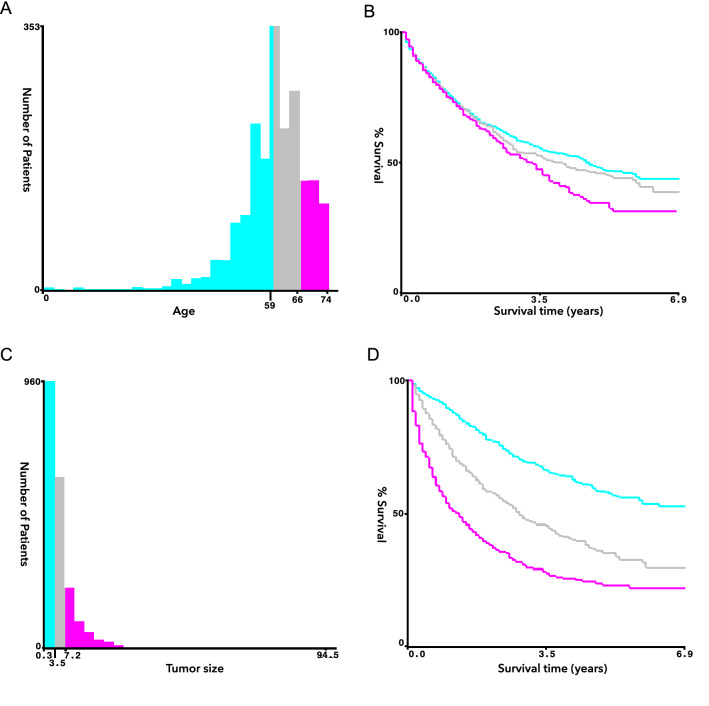
Age and tumor size were categorically divided based on the optimal cut-off value generated. (**A**) Survival histogram of age at diagnosis. (**B**) KaplanMeier survival analysis of age at diagnosis. (**C**) Survival histogram of tumor size. (**D**) KaplanMeier survival analysis of tumor size.

Age and tumor size were categorically divided based on the optimal cut-off value generated by X-tile software version 3.6.1 (Yale University School of Medicine, US) (Fig. 2). Categorical variables were expressed as a number (percent, %) and compared by the chi-square test. The propensity score matching analysis was completed by using the “Matchlt” package on R software. After obtaining PSM matched data, we analyzed the statistical differences in variables between the two groups by using the “CBCgrps” package on R software. Cancer-specific death and death of other causes were regarded as the two competing endpoint events, and the associations between variables and the risk of cancer-specific death were evaluated by Fine and Gray’s competing risk analysis. The Kaplan–Meier method and log-rank test were used for survival analysis. The independent predictive factors in the Fine and Gray competing risk model were incorporated to predict the 1-, 3-, and 5-year CSS probability. The independent risk factors were identified by univariate and multivariate Cox proportional-hazards regression analyses for OS and CSS. SPSS version 24 (SPSS, Inc., Chicago, IL, USA) and R software version 4.1.1 (R Project, Vienna, Austria) was used for all analysis. A statistically significant cutoff value was set up as P < 0.05, two-sided. P < 0.2 was selected as filter value for univariate to multivariate analysis.

## Results

### Patients' demographics and clinicopathological characteristics

A total of 1988 patients were selected in the process and randomly divided into non-chemotherapy group and chemotherapy group before propensity score matching, of which 758 received chemotherapy while the others did not. Of all the risk factors, race, AJCC stage, grade, tumor size, AFP, fibrosis score, radiotherapy, surgery and survival time were statistically different between the two groups. In the chemotherapy group, the patients who were males, Whites, age between 66 and 74 years old were more than that in the non-chemotherapy group. Besides, more patients had AJCC II or III stage, well-differentiated tumors, positive AFP, severe fibrosis and cirrhosis and tumor size between 3.5 and 7.2 cm in the chemotherapy group. And more patients with chemotherapy received radiotherapy, hepatectomy and transplantation, and didn’t undergo the surgery (S1).

### Patients' demographics and clinicopathological characteristics after propensity score matching

1092 patients were selected in the process and randomly divided into non-chemotherapy group and chemotherapy group at a ratio of 1:1, of which 546 received chemotherapy while the others did not. Of all the patients, most of them were males (79%), Whites (68%), age less than 59 years old (46%) and had tumors with AJCC I stage (39%), moderate differentiation (50%) and tumor size less than 3.5 cm (46%). In addition, the majority of patients had positive AFP (70%) and severe fibrosis and cirrhosis (77%). As for treatment, 993 (91%) didn’t receive radiotherapy and 444 (41%) were not available for any surgical procedures. The median survival time was 23 months in all 1092 patients, and 22 months, 23 months in non-chemotherapy and chemotherapy groups, respectively (Table 1).

**Table 1:** Patients' demographics and clinicopathological characteristics.

Characteristics	Total	Non-chemotherapy	Chemotherapy	p value	SMD
	(n = 1092)	(n = 546)	(n = 546)		
**Age at diagnosis, n (%)**				0.425	0.079
< 59 years	504 (46)	260 (47)	244 (45)		
59–66 years	347 (32)	174 (32)	173 (32)		
66–74 years	241 (22)	112 (21)	129 (23)		
**Sex, n (%)**				0.882	0.013
Female	229 (21)	113 (21)	116 (21)		
Male	863 (79)	433 (79)	430 (79)		
**Race, n (%)**				0.972	0.014
White	736 (68)	369 (68)	367 (67)		
Black	155 (14)	78 (14)	77 (14)		
Other	201 (18)	99 (18)	102 (19)		
**AJCC, n (%)**				0.675	0.075
I	426 (39)	207 (38)	219 (40)		
II	389 (36)	204 (38)	185 (34)		
III	185 (17)	89 (16)	96 (18)		
IV	92 (8)	46 (8)	46 (8)		
**Grade, n (%)**				0.823	0.058
Well differentiated	346 (32)	171 (31)	175 (32)		
Moderately differentiated	551 (50)	272 (50)	279 (51)		
Poorly differentiated	183 (17)	96 (18)	87 (16)		
Undifferentiated	12 (1)	7 (1)	5 (1)		
**Tumor size, n (%)**				0.889	0.029
< 3.5 cm	504 (46)	255 (46)	249 (46)		
3.5–7.2 cm	368 (34)	184 (34)	184 (33)		
> 7.2 cm	220 (20)	107 (20)	113 (21)		
**AFP, n (%)**				0.429	0.052
Negative	331 (30)	159 (29)	172 (32)		
Positive	761 (70)	387 (71)	374 (68)		
**Fibrosis score, n (%)**				0.667	0.03
F0	253 (23)	130 (24)	123 (23)		
F1	839 (77)	416 (76)	423 (77)		
**Radiotherapy, n (%)**				1	0.006
No	993 (91)	496 (91)	497 (91)		
Yes	99 (9)	50 (9)	49 (9)		
**Surgery, n (%)**				0.914	0.044
No surgery	444 (41)	219 (40)	225 (41)		
Hepatectomy	213 (20)	108 (20)	105 (19)		
Hepatectomy and transplant	298 (27)	153 (28)	145 (27)		
Others	137 (12)	66 (12)	71 (13)		
Survival times, Median (IQR)	23.00 (11.00, 45.00)	22 (7.00, 46.75)	23.00 (13.00, 43.00)	0.017*	

*Two-sided P values < 0.05.

**Two-sided P values < 0.01.

***Two-sided P values < 0.001.

AJCC, American Joint Committee on Cancer (7th).

F0, fibrosis score 0–4, non to moderate fibrosis; F1, fibrosis score 5–6, severe fibrosis and cirrhosis.

IQR, Inter-Quartile Range.

### Univariate and multivariate analysis of OS in the patients with chemotherapy and without chemotherapy

Univariate analysis demonstrated that AJCC stage, tumor size, grade, surgery and radiotherapy were associated with OS in the non-chemotherapy group, and AJCC stage, tumor size, AFP, fibrosis score, grade, surgery and radiotherapy in the chemotherapy group.

Multivariate Cox analysis of OS showed that patients with radiation had a longer OS (hazard ratio (HR), 0.603; 95% confidence interval (CI) 0.410–0.885; p = 0.010) in the non-chemotherapy group, as for those with chemotherapy, radiation made no difference to the OS (p = 0.195). AFP positive was associated with a shorter OS in the chemotherapy group (HR, 1.434; 95% CI 1.081–1.901; p = 0.012). While for patients without chemotherapy, whether AFP positive or not had no effect on the OS. Using well-differentiated grade as a reference, poorly differentiated tumors (HR, 1.947; 95% CI 1.349–2.809; p = < 0.001) and undifferentiated tumors (HR, 3.892; 95% CI 1.496–10.128; p = 0.005) were associated with poor OS in the non-chemotherapy group, while only poorly differentiated tumors (HR, 1.578; 95% CI 1.097–2.269; p = 0.014) in the chemotherapy group.

In both groups, AJCC TNM stage (II, III, IV), tumor size (3.5–7.2 cm, > 7.2 cm) and surgical options (hepatectomy, hepatectomy and transplant, others) were identified as significantly associated with OS in multivariate analysis (Table 2).

**Table 2:** Univariate and multivariate analysis of OS in the patients with chemotherapy and without chemotherapy.

Variables	Non-chemotherapy(n = 546)				Chemotherapy(n = 546)			
		Univariate analysis	Multivariate analysis			Univariate analysis	Multivariate analysis	
	N	P value	HR(95%CI)	P value	N	P value	HR(95%CI)	P value
**Sex**								
Female	113		Reference		116		—	
Male	433	0.0772	0.847 (0.610–1.175)	0.320	430	0.430	—	—
**Age at diagnosis, n (%)**								
< 59 years	260		Reference		244		Reference	
59–66 years	174	0.0954	1.229 (0.917–1.649)	0.168	173	0.390	1.158 (0.875–1.533)	0.306
66–74 years	112	0.9629	1.032 (0.755–1.409)	0.844	129	0.160	1.175 (0.860–1.607)	0.312
**Race**								
White	369		—		367		—	
Black	78	0.772	—	—	77	0.460	—	—
Others	99	0.788	—	—	102	0.510	—	—
**AJCC**								
I	207		Reference		219		Reference	
II	204	0.357	1.646 (1.191–2.276)	0.002**	185	0.748	1.356 (0.992–1.855)	0.057
III	89	< 0.001***	2.502 (1.729–3.620)	< 0.001***	96	< 0.001***	1.809 (1.264–2.587)	0.001**
IV	46	< 0.001***	3.326 (2.203–5.021)	< 0.001***	46	< 0.001***	2.479 (1.604–3.833)	< 0.001***
**Tumor size**								
< 3.5 cm	255		Reference		249		Reference	
3.5–7.2 cm	184	< 0.001***	1.359 (0.986–1.872)	0.061	184	< 0.001***	1.523 (1.119–2.072)	0.008**
> 7.2 cm	107	< 0.001***	1.989 (1.337–2.957)	< 0.001***	113	< 0.001***	2.220 (1.496–3.294)	< 0.001***
**AFP**								
Negative	159		Reference		172		Reference	
Positive	387	0.186	1.038 (0.784–1.375)	0.793	374	0.0499*	1.434 (1.081–1.901)	0.012*
**Fibrosis score**								
F0	130		—		123		Reference	
F1	416	0.358	—	—	423	0.046*	1.066 (0.780–1.458)	0.69
**Grade**								
Well differentiated	171		Reference		175		Reference	
Moderately differentiated	272	0.0941	1.466 (1.092–1.967)	0.011*	279	0.980	1.226 (0.922–1.630)	0.161
Poorly differentiated	96	< 0.001***	1.947 (1.349–2.809)	< 0.001***	87	0.00756**	1.578 (1.097–2.269)	0.014*
Undifferentiaed	7	0.045*	3.892 (1.496–10.128)	0.005**	5	0.246	1.732 (0.604–4.968)	0.307
**Surgery**								
No surgery	219		Reference		225		Reference	
Hepatectomy	108	< 0.001***	0.151 (0.100–0.228)	< 0.001***	105	< 0.001***	0.361 (0.254–0.514)	< 0.001***
Hepatectomy and transplant	153	< 0.001***	0.122 (0.078–0.190)	< 0.001***	145	< 0.001***	0.154 (0.098–0.242)	< 0.001***
Others	66	< 0.001***	0.431 (0.286–0.650)	< 0.001***	71	0.004	0.681 (0.479–0.968)	0.032*
**Radiotherapy**								
No	496		Reference		497		Reference	
Yes	50	< 0.001***	0.603 (0.410–0.885)	0.010**	49	< 0.001***	1.288 (0.878–1.887)	0.195

*Two-sided P values < 0.05.

**Two-sided P values < 0.01.

***Two-sided P values < 0.001.

OS, overall survival; HR, hazard ratio; CI, coindidence intervals; AJCC, American Joint Committee on Cancer (7th).

F0, fibrosis score 0–4, non to moderate fibrosis; F1, fibrosis score 5–6, severe fibrosis and cirrhosis.

### Univariate and multivariate analysis of CSS in the patients with chemotherapy and without chemotherapy

Univariate analysis demonstrated that AJCC stage, tumor size, grade, surgery and radiotherapy were associated with CSS in the non-chemotherapy group, and AJCC stage, tumor size, AFP, fibrosis score, grade, surgery and radiotherapy in the chemotherapy group.

Multivariate Cox analysis of CSS showed that patients with radiation had a longer CSS (hazard ratio (HR),0.519; 95% CI 0.341–0.789; p = 0.002) in the non-chemotherapy group, while for those with chemotherapy, radiation made no difference to the CSS (p = 0.429). AFP positive was associated with a shorter OS in the chemotherapy group (HR, 1.487; 95% CI 1.095–2.020; p = 0.011). While for patients without chemotherapy, whether AFP positive or not did not affect CSS. All surgical options were associated with a longer CSS in the non-chemotherapy group, but in the chemotherapy group, other surgical therapies were not associated with CSS (p = 0.051). Using well-differentiated grade as a reference, poorly differentiated tumors and undifferentiated tumors were associated with poor CSS in the non-chemotherapy group, while only poorly differentiated tumors in the chemotherapy group.

In both groups, AJCC TNM stage (III, IV) and tumor size (3.5–7.2 cm, > 7.2 cm) were identified as significantly associated with the CSS in multivariate analysis (Table 3). The conclusion above was consistent with the result of competing risk regression analysis (S3).

**Table 3:** Univariate and multivariate analysis of CSS in the patients with chemotherapy and without chemotherapy.

Variables	Non-chemotherapy(n = 503)				Chemotherapy(n = 509)			
		Univariate analysis	Multivariate analysis			Univariate analysis	Multivariate analysis	
	N	P value	HR(95%CI)	P value	N	P value	HR(95%CI)	P value
**Sex**								
Female	106		Reference		110		Reference	
Male	397	0.0904	0.757 (0.528–1.086)	0.131	399	0.098	0.094 (0.709–1.310)	0.814
**Age at diagnosis**								
< 59 years	237		Reference		231		Reference	
59–66 years	160	0.9301	1.294 (0.935–1.791)	0.121	160	0.1412	1.154 (0.855–1.557)	0.351
66–74 years	106	0.0441*	1.083 (0.775–1.515)	0.640	118	0.0198*	1.108 (0.791–1.553)	0.550
**Race**								
White	338		—		342		—	
Black	70	0.943	—	—	70	0.371	—	—
Others	95	0.992	—	—	97	0.596	—	—
**AJCC**								
I	191		Reference		202		Reference	
II	188	0.255	1.995 (1.383–2.876)	< 0.001*	173	0.598	1.506 (1.066–2.126)	0.020*
III	79	< 0.001***	2.623 (1.752–3.929)	< 0.001*	91	< 0.001***	1.968 (1.346–2.878)	< 0.001***
IV	45	< 0.001***	3.791 (2.452–5.859)	< 0.001*	43	< 0.001***	2.589 (1.630–3.777)	< 0.001***
**Tumor size**								
< 3.5 cm	228		Reference		232		Reference	
3.5–7.2 cm	176	< 0.001***	1.663 (1.165–2.373)	0.005**	168	< 0.001***	1.574 (1.121–2.210)	0.009**
> 7.2 cm	99	< 0.001***	2.274 (1.470–3.516)	< 0.001*	109	< 0.001***	2.481 (1.630–3.777)	< 0.001***
**AFP**								
Negative	141		Reference		160		Reference	
Positive	362	0.0524	1.173 (0.854–1.613)	0.325	349	0.0433*	1.487 (1.095–2.020)	0.011*
**Fibrosis score**								
F0	122		—		119		Reference	
F1	381	0.49	—	—	390	0.0252*	1.105 (0.796–1.534)	0.550
**Grade, n (%)**								
Well differentiated	156		Reference		163		Reference	
Moderately differentiated	250	0.0487*	1.522 (1.095–2.117)	0.013*	256	0.76322	1.241 (0.908–1.696)	0.176
Poorly differentiated	93	< 0.001***	2.097 (1.412–3.114)	< 0.001***	85	0.00244**	1.616 (1.104–2.365)	0.014*
Undifferentiated	4	0.3476	2.911 (0.656–12.913)	0.160	5	0.18377	1.792 (0.618–5.195)	0.282
**Surgery**								
No surgery	203		Reference		202		Reference	
Hepatectomy	101	< 0.001***	0.124 (0.078–0.198)	< 0.001***	103	< 0.001***	0.357 (0.248–0.513)	< 0.001***
Hepatectomy and transplant	139	< 0.001***	0.074 (0.042–0.132)	< 0.001***	137	< 0.001***	0.111 (0.064–0.192)	< 0.001***
Others	60	< 0.001***	0.393 (0.249–0.620)	< 0.001***	67	< 0.001***	0.689 (0.474–1.001)	0.051
**Radiotherapy**								
No	458		Reference		465		Reference	
Yes	45	< 0.001***	0.519 (0.341–0.789)	0.002**	44	< 0.001***	1.182 (0.781–1.789)	0.429

*Two-sided P values < 0.05.

**Two-sided P values < 0.01.

***Two-sided P values < 0.001.

CSS, cancer-specific survival; HR, hazard ratio; CI, coindidence intervals; AJCC, American Joint Committee on Cancer (7th).

F0, fibrosis score 0–4, non to moderate fibrosis; F1, fibrosis score 5–6, severe fibrosis and cirrhosis.

### Cumulative cancer-specific mortality and other causes-specific mortality of patients with HCC stratified for chemotherapy

Table 4 showed the cumulative incidence of cancer-specific death increased with elevated AJCC TNM stage, poorly differentiated grade, increasing tumor size, positive AFP, radiotherapy and surgery in both groups. In addition, in the chemotherapy group, severe fibrosis or cirrhosis was associated with the increased cumulative incidence of cancer-specific death (p = 0.007), but not in the non-chemotherapy group (p = 0.638) (Table 4).

**Table 4:** Cumulative cancer-specific mortality and other causes-specific mortality of patients with HCC stratified for chemotherapy.

Variables	Non-chemotherapy								Chemotherapy							
	Cancer-specific mortality				Other causes-specific mortality				Cancer-specific mortality				Other causes-specific mortality			
	1-year	3-year	5-year	P value	1-year	3-year	5-year	P value	1-year	3-year	5-year	P value	1-year	3-year	5-year	P value
**Sex**				0.137				0.489				0.082				0.443
Female	0.231	0.337	0.394		0.036	0.065	0.065		0.251	0.485	0.557		0.000	0.044	0.091	
Male	0.278	0.422	0.484		0.046	0.078	0.091		0.187	0.415	0.503		0.030	0.066	0.082	
**Age at diagnosis**				0.057				0.633				0.149				0.304
< 59 years	0.254	0.379	0.444		0.054	0.077	0.097		0.177	0.381	0.486		0.024	0.043	0.067	
59–66 years	0.255	0.394	0.413		0.041	0.086	0.086		0.227	0.454	0.520		0.023	0.065	0.087	
66–74 years	0.323	0.481	0.603		0.027	0.054	0.054		0.210	0.498	0.573		0.023	0.091	0.110	
**Race**				0.931				0.285				0.713				0.535
White	0.265	0.399	0.472		0.044	0.079	0.090		0.203	0.430	0.516		0.025	0.062	0.078	
Black	0.269	0.377	0.421		0.052	0.095	0.117		0.208	0.473	0.574		0.026	0.091	0.123	
Others	0.283	0.451	0.469		0.040	0.040	0.040		0.187	0.394	0.459		0.020	0.034	0.074	
**AJCC**				< 0.001***				0.301				< 0.001***				0.895
I	0.160	0.266	0.316		0.049	0.079	0.092		0.106	0.317	0.416		0.018	0.072	0.091	
II	0.158	0.322	0.385		0.025	0.070	0.078		0.120	0.364	0.458		0.022	0.056	0.088	
III	0.494	0.623	0.708		0.101	0.101	0.123		0.419	0.678	0.738		0.021	0.045	0.060	
IV	0.804	0.953	NA		0.000	0.022	NA		0.522	0.714	0.714		0.065	0.065	0.065	
**Tumor size**				< 0.001***				0.086				< 0.001***				0.206
< 3.5 cm	0.110	0.196	0.268		0.051	0.098	0.112		0.061	0.273	0.349		0.016	0.056	0.099	
3.5–7.2 cm	0.329	0.546	0.613		0.022	0.047	0.047		0.214	0.470	0.604		0.039	0.089	0.089	
> 7.2 cm	0.543	0.677	0.699		0.066	0.066	0.085		0.488	0.709	0.738		0.018	0.028	0.042	
**AFP**				0.019				0.056				0.047				0.892
Positive	0.296	0.428	0.494		0.034	0.062	0.067		0.229	0.469	0.524		0.024	0.063	0.077	
Negative	0.202	0.344	0.393		0.070	0.107	0.131		0.140	0.349	0.501		0.023	0.057	0.098	
**Fibrosis score**				0.638				0.441				0.007**				0.101
F0	0.239	0.377	0.450		0.031	0.049	0.064		0.264	0.525	0.638		0.033	0.033	0.033	
F1	0.277	0.412	0.469		0.048	0.083	0.092		0.183	0.404	0.482		0.021	0.068	0.097	
**Grade, n (%)**				< 0.001***				0.0012**				< 0.001***				0.203
Well differentiated	0.188	0.284	0.374		0.047	0.084	0.094		0.156	0.394	0.519		0.023	0.064	0.079	
Moderately differentiated	0.244	0.413	0.465		0.041	0.074	0.089		0.180	0.393	0.438		0.032	0.075	0.105	
Poorly differentiated	0.480	0.599	0.638		0.032	0.032	0.032		0.346	0.595	0.712		0.000	0.013	0.031	
Undifferentiated	0.286	0.286	NA		0.286	0.500	NA		0.400	0.800	0.800		0.000	0.000	0.000	
**Surgery**				< 0.001***				0.927				< 0.001***				0.058
No surgery	0.549	0.724	0.816		0.055	0.069	0.077		0.345	0.611	0.679		0.040	0.079	0.123	
Hepatectomy	0.113	0.272	0.311		0.038	0.063	0.083		0.202	0.437	0.557		0.010	0.020	0.020	
Hepatectomy and transplant	0.026	0.083	0.115		0.046	0.074	0.087		0.028	0.115	0.130		0.021	0.054	0.064	
Others	0.155	0.353	0.438		0.016	0.117	0.117		0.100	0.483	0.773		0.000	0.073	0.073	
**Radiotherapy**				0.017*				0.474				< 0.001***				0.180
No	0.263	0.384	0.447		0.045	0.071	0.083		0.186	0.412	0.495		0.020	0.056	0.080	
Yes	0.323	0.639	0.679		0.041	0.125	0.125		0.352	0.648	0.809		0.063	0.110	0.110	

*Two-sided P values < 0.05.

**Two-sided P values < 0.01.

***Two-sided P values < 0.001.

AJCC, American Joint Committee on Cancer (7th).

F0, fibrosis score 0–4, non to moderate fibrosis; F1, fibrosis score 5–6, severe fibrosis and cirrhosis.

**Table 5:** Comparision of causes of death in HCC patients with chemotherapy and without chemotherapy.

COD, n (%)	Total(n = 547)	Non-chemotherapy(n = 272)	Chemotherapy(n = 275)	Odds ratio(95%CI)	P
Liver related	426(78)	209(77)	217(79)	0.887(0.592–1.328)	0.56
Miscellaneous Malignant	22(4)	11(4)	11(4)	1.011(0.431–2.374)	0.979
Cardiovascular disease	10(2)	6(2)	4(2)	1.528(0.426–5.477)	0.515
Lung injury	8(1)	5(2)	3(1)	1.698(0.402–7.175)	0.472
Infections	33(6)	18(7)	15(5)	1.228(0.606–2.490)	0.568
Others	48(9)	23(8)	25(9)	0.966(0.531–1.757)	0.91
Total					0.939

*Two-sided P values < 0.05.

**Two-sided P values < 0.01.

***Two-sided P values < 0.001.

COD, cause of death.

**Table 6:** Cumulative cancer-specific death and other causes-specific death of patients with HCC between the non-chemotherapy group and chemotherapy group.

Variables	Cancer-specific death				Other causes-specific death			
	1-year	3-year	5-year	P value	1-year	3-year	5-year	P value
**Chemotherapy**				0.819				0.459
No	0.268	0.404	0.465		0.044	0.075	0.086	
Yes	0.201	0.431	0.515		0.024	0.061	0.083	

### The cumulative incidence and causes of death between the non-chemotherapy and chemotherapy groups

The cumulative incidence of cancer-specific death was higher than that of other causes-specific death, but there was no statistically significant difference between the two groups. Compared with the non-chemotherapy group, the 1-year cumulative incidence of cancer-specific death was decreased in the chemotherapy group, but the 3-year and 5-year cumulative incidence were increased (Table 5). What’s more, the 1-year, 3-year and 5-year cumulative incidence of other causes-specific death were all decreased after receiving chemotherapy (Table 6).

In both groups, the majority of patients died of liver-related diseases. But the significant differences in causes of death between the two groups were not found (Table 5).

### Patients’ demographics and clinicopathological characteristics grouped by AJCC stage

Considering the different severity of the disease, clinicians were inclined to take different treatment options, so we divided the patients into groups according to the AJCC stage. Among the total 1092 patients, 426 were in AJCC I stage, 389 in II, 185 in III, and 92 in IV. Regardless of the AJCC stage, patients with well-differentiated and moderately differentiated tumors formed a sizeable majority. Overall, the degree of tumor differentiation decreased with the increasing AJCC stage. Tumor size, fibrosis score and AFP varied greatly with different stages. And surgery alone (S) or surgery combined with chemotherapy (SC) was the major treatment for patients in AJCC I (S, 31%; SC, 31%) and AJCC II stage(S, 39%; SC, 32%). While in AJCC III and IV stage, chemotherapy alone (C) (AJCC III, 23%; AJCC IV, 21%) or surgery combined with chemotherapy (SC) (AJCC III, 20%; AJCC IV, 14%) was the main treatment for patients (S2).

### Competing risk analysis of cancer-specific death in HCC patients grouped by AJCC stage

Competing risk analysis of cancer-specific death in HCC patients grouped by AJCC stage showed that surgery alone (S) was the better treatment option than surgery combined with chemotherapy (SC) in AJCC I, II and III stage, but not in AJCC IV stage. This conclusion was broadly consistent with the results obtained by grouping chemotherapy. The contrary result in the AJCC IV stage may be due to the fact that most of the patients at this stage chose the surgery combined with chemotherapy, while very few chose the surgery alone (S3).

## Discussion

Cancer-specific death and other causes-specific death are mutually exclusive endpoints in oncology research. Competing events are regarded as censoring and cancer-specific mortalities, which may be overestimated when using traditional Kaplan–Meier and Cox methods^23,24^. Therefore, there may be a deviation in the prognosis assessment of patients, which will create a substantial psychological burden to patients and affect their lives. Currently, we conducted a real-world study based on the SEER database to identify the independent predictive factors of CSS of patients diagnosed with HCC depending on whether receiving chemotherapy or not through using the competing risk method. And we used the competing risk model to predict cumulative mortality. By comparing the Cox regression method and competing risk method, we could find the more precise predictor for the prognosis of HCC patients.

The two regression models both identified AJCC stage, tumor size, grade, surgical therapy and radiotherapy as independent predictive factors of OS and CSS in the non-chemotherapy group, and AJCC stage, tumor size, grade, surgical therapy as well as AFP in the chemotherapy group. In our research, competing factors had little impact on the mortality of these patients. Regardless of chemotherapy, higher AJCC stage and grade, larger tumor size and without surgical procedures were associated with poor prognosis. In addition, radiotherapy had been proved to be an effective treatment for patients with unresectable HCC^25^, in this research, radiotherapy was also an effective treatment for patients who hadn’t received chemotherapy. And negative AFP predicted a longer survival time for those who had received chemotherapy, which was consistent with many studies^26,27^.

Through comparing the prognosis outcomes of different surgical therapies, we found that liver transplantation was the most effective treatment regardless of chemotherapy. And the proportion of patients dead of HCC after liver transplantation was similar in both groups. Of 153 patients in the non-chemotherapy group were administered liver transplantation and 30 died during follow-up, including 16 (53%) of HCC and 14 (47%) of other causes. While in the chemotherapy group, of 145 patients, 24 died, including 16 (67%) of HCC and 8 (33%) of other causes. Thus, the survival analysis of patients receiving liver transplantation should consider the competing events. Interestingly, for patients with hepatectomy or transplantation, the cumulative incidence of cancer-specific death was increased in the chemotherapy group, but decreased for patients without any surgical therapies after being administered chemotherapy. In addition, for patients undergoing other types of surgery and chemotherapy, the 1-year mortality rate was decreased, but the 3-year and 5-year mortality rates were increased. That was to say, our research demonstrated that transplantation remained the optimal treatment choice, but for patients who had received surgery, the systematic chemotherapy was not conducive to the long-term survival. However, this conclusion remains controversial. Several studies had shown that the combination of surgical treatment and chemotherapy was of benefit for patients with HCC^12,13^. Whereas, neither local^14,15^ nor systemic^16–18^ chemotherapy studies had been able to show the advantage of adjuvant chemotherapy in the prognosis of HCC patients who had undergone surgery. Therefore, this needs to be studied further, and considers the individual differences and diversity of chemotherapies, as well as the severity of the disease, such as the presence of thrombogenesis and distant metastasis. Considering the different severity of the disease, clinicians were inclined to take different treatments, so we divided the patients into groups according to AJCC stage. The conclusion was broadly consistent with the results obtained by grouping chemotherapy except in the AJCC IV stage. But the contrary result in the AJCC IV stage may be due to the fact that most of the patients at this stage chose the surgery combined with chemotherapy, while very few chose the surgery alone.

Liver resection or transplantation is not appropriate for patients with inferior vena cava thrombosis (IVTT) or portal venous tumor thrombosis (PVTT), but radiotherapy can be a treatment option in order to shrink the vascular thrombus. Researches have shown that the combination of radiotherapy and transcatheter arterial chemoembolization (TACE) was better for patients’ survival than TACE alone^19–21^. However, according to our multivariate analysis, radiotherapy was beneficial to the survival of patients without chemotherapy, while for patients with chemotherapy, radiotherapy had no effect on the overall survival. Besides, the cumulative incidence of death of HCC and other causes were increased for patients when administered radiotherapy in both groups. This result may be due to the fact that our cumulative death probability analysis was univariate, or it may be associated with adverse reactions of radiotherapy, such as radiation-induced liver disease (RILD)^22^.

Although using population-based data from SEER can reduce selection or treatment biases, we can’t ignore its limitations. First, the data sources were limited to the United States. Different countries could have diverse characteristics of diseases and options of treatments. Second, the SEER database provided only macroscopic information about the treatment, which lacked individual information about it. Additionally, it was a retrospective study. Finally, the database only contained a baseline lacking the dynamic changes in each indicator during follow-up, which led to the limitations of our research.

### Method statement

All methods were carried out in accordance with relevant guidelines and regulations.

## Supplementary Information

Supplementary Information 1.

Supplementary Information 2.

Supplementary Information 3.

## Abbreviations

HCC: Hepatocellular carcinoma
ICC: Intrahepatic cholangiocarcinoma
AASLD: The American Association for the Study of Liver Diseases
RFA: Heat-Radio-Frequency ablation
PEI: Percutaneous Ethanol Injection
SEER: The Surveillance, Epidemiology, and End Results
PSM: Propensity score matching
OS: Overall survival
CSS: Cancer-specific survival
CI: Coincidence interval
AJCC: American Joint Committee on Cancer (7th)
HR: Hazard ratio
COD: Cause of death
RILD: Radiation-induced liver disease
