# Publisher Correction: Association between chemotherapy and prognostic factors of survival in hepatocellular carcinoma: a SEER population-based cohort study

**DOI:** 10.1038/s41598-022-05396-4

**Published:** 2022-01-31

**Authors:** Meiqi Liu, Mengying Xu, Tiantian Tang

**Affiliations:** 1 Department and Institute of Infectious Disease, Xi’an Children’s Hospital, Xi’an Jiaotong University, Xi’an, 710003 China; 2 Department of Cardiology, Guangzhou Women and Children Medical Center, Guangzhou, China

Correction to: *Scientific Reports* https://doi.org/10.1038/s41598-021-02698-x, published online 09 December 2021

The original version of this Article contained extensive errors in Tables 1, 2, 3, and 5, where column headings were mislabelled and data was listed in the incorrect columns.

The original Tables 1, 2, 3 and 5 and accompanying legends appear below.

**Table 1:** Patients’ demographics and clinicopathological characteristics.

Total	Non-chemotherapy	Chemotherapy	Characteristics	p value	SMD
(n = 1092)	(n = 546)				(n = 546)
**Age at diagnosis, n (%)**	0.425				0.079
< 59 years	504 (46)		260 (47)		244 (45)
59–66 years	347 (32)		174 (32)		173 (32)
66–74 years	241 (22)		112 (21)		129 (23)
**Sex, n (%)**		0.882		0.013	
Female	229 (21)		113 (21)		116 (21)
Male	863 (79)		433 (79)		430 (79)
**Race, n (%)**		0.972		0.014	
White	736 (68)		369 (68)		367 (67)
Black	155 (14)		78 (14)		77 (14)
Other	201 (18)		99 (18)		102 (19)
**AJCC, n (%)**		0.675		0.075	
I	426 (39)		207 (38)		219 (40)
II	389 (36)		204 (38)		185 (34)
III	185 (17)		89 (16)		96 (18)
IV	92 (8)		46 (8)		46 (8)
**Grade, n (%)**		0.823		0.058	
Well differentiated	346 (32)		171 (31)		175 (32)
Moderately differentiated	551 (50)		272 (50)		279 (51)
Poorly differentiated	183 (17)		96 (18)		87 (16)
Undifferentiated	12 (1)		7 (1)		5 (1)
**Tumor size, n (%)**		0.889		0.029	
< 3.5 cm	504 (46)		255 (46)		249 (46)
3.5–7.2 cm	368 (34)		184 (34)		184 (33)
> 7.2 cm	220 (20)		107 (20)		113 (21)
**AFP, n (%)**		0.429		0.052	
Negative	331 (30)		159 (29)		172 (32)
Positive	761 (70)		387 (71)		374 (68)
**Fibrosis score, n (%)**		0.667		0.03	
F0	253 (23)		130 (24)		123 (23)
F1	839 (77)		416 (76)		423 (77)
**Radiotherapy, n (%)**		1		0.006	
No	993 (91)		496 (91)		497 (91)
Yes	99 (9)		50 (9)		49 (9)
**Surgery, n (%)**		0.914		0.044	
No surgery	444 (41)		219 (40)		225 (41)
Hepatectomy	213 (20)		108 (20)		105 (19)
Hepatectomy and transplant	298 (27)		153 (28)		145 (27)
Others	137 (12)		66 (12)		71 (13)
Survival times, Median (IQR)	23.00 (11.00, 45.00)		22 (7.00, 46.75)	23.00 (13.00, 43.00)	0.017*

*Two-sided P values < 0.05. **Two-sided P values < 0.01. ***Two-sided P values < 0.001. AJCC, American Joint Committee on Cancer (7th). F0, fibrosis score 0–4, non to moderate fibrosis; F1, fibrosis score 5–6, severe fibrosis and cirrhosis. IQR, Inter-Quartile Range.

**Table 2:** Univariate and multivariate analysis of OS in the patients with chemotherapy and without chemotherapy.

Variables			Non-chemotherapy(n = 546)			Chemotherapy(n = 546)		
Univariate analysis			Univariate analysis		Multivariate analysis		Multivariate analysis	
N		P value	HR(95%CI)	P value	N	P value	HR(95%CI)	P value
**Sex**								
Female	113		Reference		116		—	
Male	433	0.0772	0.847 (0.610–1.175)	0.320	430	0.430	—	—
**Age at diagnosis, n (%)**								
< 59 years	260		Reference		244		Reference	
59–66 years	174	0.0954	1.229 (0.917–1.649)	0.168	173	0.390	1.158 (0.875–1.533)	0.306
66–74 years	112	0.9629	1.032 (0.755–1.409)	0.844	129	0.160	1.175 (0.860–1.607)	0.312
**Race**								
White	369		—		367		—	
Black	78	0.772	—	—	77	0.460	—	—
Others	99	0.788	—	—	102	0.510	—	—
**AJCC**								
I	207		Reference		219		Reference	
II	204	0.357	1.646 (1.191–2.276)	0.002**	185	0.748	1.356 (0.992–1.855)	0.057
III	89	< 0.001***	2.502 (1.729–3.620)	< 0.001***	96	< 0.001***	1.809 (1.264–2.587)	0.001**
IV	46	< 0.001***	3.326 (2.203–5.021)	< 0.001***	46	< 0.001***	2.479 (1.604–3.833)	< 0.001***
**Tumor size**								
< 3.5 cm	255		Reference		249		Reference	
3.5–7.2 cm	184	< 0.001***	1.359 (0.986–1.872)	0.061	184	< 0.001***	1.523 (1.119–2.072)	0.008**
> 7.2 cm	107	< 0.001***	1.989 (1.337–2.957)	< 0.001***	113	< 0.001***	2.220 (1.496–3.294)	< 0.001***
**AFP**								
Negative	159		Reference		172		Reference	
Positive	387	0.186	1.038 (0.784–1.375)	0.793	374	0.0499*	1.434 (1.081–1.901)	0.012*
**Fibrosis score**								
F0	130		—		123		Reference	
F1	416	0.358	—	—	423	0.046*	1.066 (0.780–1.458)	0.69
**Grade**								
Well differentiated	171		Reference		175		Reference	
Moderately differenti- ated	272	0.0941	1.466 (1.092–1.967)	0.011*	279	0.980	1.226 (0.922–1.630)	0.161
Poorly differentiated	96	< 0.001***	1.947 (1.349–2.809)	< 0.001***	87	0.00756**	1.578 (1.097–2.269)	0.014*
Undifferentiaed	7	0.045*	3.892 (1.496–10.128)	0.005**	5	0.246	1.732 (0.604–4.968)	0.307
**Surgery**								
No surgery	219		Reference		225		Reference	
Hepatectomy	108	< 0.001***	0.151 (0.100–0.228)	< 0.001***	105	< 0.001***	0.361 (0.254–0.514)	< 0.001***
Hepatectomy and transplant	153	< 0.001***	0.122 (0.078–0.190)	< 0.001***	145	< 0.001***	0.154 (0.098–0.242)	< 0.001***
Others	66	< 0.001***	0.431 (0.286–0.650)	< 0.001***	71	0.004	0.681 (0.479–0.968)	0.032*
**Radiotherapy**								
No	496		Reference		497		Reference	
Yes	50	< 0.001***	0.603 (0.410–0.885)	0.010**	49	< 0.001***	1.288 (0.878–1.887)	0.195

*Two-sided P values < 0.05. **Two-sided P values < 0.01. ***Two-sided P values < 0.001. OS, overall survival; HR, hazard ratio; CI, coindidence intervals; AJCC, American Joint Committee on Cancer (7th). F0, fibrosis score 0–4, non to moderate fibrosis; F1, fibrosis score 5–6, severe fibrosis and cirrhosis.

**Table 3:** Univariate and multivariate analysis of CSS in the patients with chemotherapy and without chemotherapy.

Variables			Non-chemotherapy(n = 503)			Chemotherapy(n = 509)		
Univariate analysis			Univariate analysis		Multivariate analysis		Multivariate analysis	
	N	P value	HR(95%CI)	P value	N	P value	HR(95%CI)	P value
**Sex**								
Female	106		Reference		110		Reference	
Male	397	0.0904	0.757 (0.528–1.086)	0.131	399	0.098	0.094 (0.709–1.310)	0.814
**Age at diagnosis**								
< 59 years	237		Reference		231		Reference	
59–66 years	160	0.9301	1.294 (0.935–1.791)	0.121	160	0.1412	1.154 (0.855–1.557)	0.351
66–74 years	106	0.0441*	1.083 (0.775–1.515)	0.640	118	0.0198*	1.108 (0.791–1.553)	0.550
**Race**								
White	338		—		342		—	
Black	70	0.943	—	—	70	0.371	—	—
Others	95	0.992	—	—	97	0.596	—	—
**AJCC**								
I	191		Reference		202		Reference	
II	188	0.255	1.995 (1.383–2.876)	< 0.001*	173	0.598	1.506 (1.066–2.126)	0.020*
III	79	< 0.001***	2.623 (1.752–3.929)	< 0.001*	91	< 0.001***	1.968 (1.346–2.878)	< 0.001***
IV	45	< 0.001***	3.791 (2.452–5.859)	< 0.001*	43	< 0.001***	2.589 (1.630–3.777)	< 0.001***
**Tumor size**								
< 3.5 cm	228		Reference		232		Reference	
3.5–7.2 cm	176	< 0.001***	1.663 (1.165–2.373)	0.005**	168	< 0.001***	1.574 (1.121–2.210)	0.009**
> 7.2 cm	99	< 0.001***	2.274 (1.470–3.516)	< 0.001*	109	< 0.001***	2.481 (1.630–3.777)	< 0.001***
**AFP**								
Negative	141		Reference		160		Reference	
Positive	362	0.0524	1.173 (0.854–1.613)	0.325	349	0.0433*	1.487 (1.095–2.020)	0.011*
**Fibrosis score**								
F0	122		—		119		Reference	
F1	381	0.49	—	—	390	0.0252*	1.105 (0.796–1.534)	0.550
**Grade, n (%)**								
Well differentiated	156		Reference		163		Reference	
Moderately differenti- ated	250	0.0487*	1.522 (1.095–2.117)	0.013*	256	0.76322	1.241 (0.908–1.696)	0.176
Poorly differentiated	93	< 0.001***	2.097 (1.412–3.114)	< 0.001***	85	0.00244**	1.616 (1.104–2.365)	0.014*
Undifferentiated	4	0.3476	2.911 (0.656–12.913)	0.160	5	0.18377	1.792 (0.618–5.195)	0.282
**Surgery**								
No surgery	203		Reference		202		Reference	
Hepatectomy	101	< 0.001***	0.124 (0.078–0.198)	< 0.001***	103	< 0.001***	0.357 (0.248–0.513)	< 0.001***
Hepatectomy and transplant	139	< 0.001***	0.074 (0.042–0.132)	< 0.001***	137	< 0.001***	0.111 (0.064–0.192)	< 0.001***
Others	60	< 0.001***	0.393 (0.249–0.620)	< 0.001***	67	< 0.001***	0.689 (0.474–1.001)	0.051
**Radiotherapy**								
No	458		Reference		465		Reference	
Yes	45	< 0.001***	0.519 (0.341–0.789)	0.002**	44	< 0.001***	1.182 (0.781–1.789)	0.429

*Two-sided P values < 0.05. **Two-sided P values < 0.01. ***Two-sided P values < 0.001. CSS, cancer-specific survival; HR, hazard ratio; CI, coindidence intervals; AJCC, American Joint Committee on Cancer (7th). F0, fibrosis score 0–4, non to moderate fibrosis; F1, fibrosis score 5–6, severe fibrosis and cirrhosis.

**Table 5:** Comparision of causes of death in HCC patients with chemotherapy and without chemotherapy.

COD, n (%)	Total(n = 547)	Non-chemotherapy(n = 272)	Chemotherapy(n = 275)	Odds ratio(95%CI)	P
Liver related	426(78)	209(77)	217(79)	0.887(0.592–1.328)	0.56
Miscellaneous Malignant	22(4)	11(4)	11(4)	1.011(0.431–2.374)	0.979
Cardiovascular disease	10(2)	6(2)	4(2)	1.528(0.426–5.477)	0.515
Lung injury	8(1)	5(2)	3(1)	1.698(0.402–7.175)	0.472
Infections	33(6)	18(7)	15(5)	1.228(0.606–2.490)	0.568
Others	48(9)	23(8)	25(9)	0.966(0.531–1.757)	0.91
Total			0.939		

*Two-sided P values < 0.05. **Two-sided P values < 0.01. ***Two-sided P values < 0.001. COD, cause of death.

The original Article has been corrected.

