# Supplementary material for: Association between chemotherapy and prognostic factors of survival in hepatocellular carcinoma: a SEER population-based cohort study
Source: Sci Rep. 2021 Dec 9;11:23754. doi: 10.1038/s41598-021-02698-x (PMC8660869; doi:10.1038/s41598-021-02698-x)
Supplement: Supplementary file 1 — Supplementary Information 1. [file 41598_2021_2698_MOESM1_ESM.pdf]

S1. Patients' demographics and clinicopathological characteristics before propensity score matching

Characteristics	Total (n = 1988)	Non-chemotherapy (n=1230)	Chemotherapy (n=758)	p value
Age at diagnosis, n (%)				0.68
<59 years	863 (43)	535 (43)	328 (43)	
59-66 years	633 (32)	311 (25)	181 (24)	
66-74 years	492 (25)	112(21)	129(23)	
Sex, n (%)				0.294
Female	419 (21)	269 (22)	150 (20)	
Male	1569 (79)	961 (78)	608 (80)	
Race, n (%)				< 0.001***
White	1309 (66)	774 (63)	535 (71)	
Black	274 (14)	175 (14)	99 (13)	
Other	405 (20)	281 (23)	124 (16)	
AJCC, n (%)				< 0.001***
I	941 (47)	692 (56)	249 (33)	
II	606 (30)	347 (28)	259 (34)	
III	299 (15)	136 (11)	163 (22)	
IV	142 (7)	55 (4)	87 (11)	
Grade, n (%)				0.016*
Well differentiated	610 (31)	348 (28)	262 (35)	
Moderately differentiated	1016 (51)	644 (52)	372 (49)	
Poorly differentiated	342 (17)	227 (18)	115 (15)	
Undifferentiated	20 (1)	11 (1)	9 (1)	
Tumor size, n (%)				< 0.001***
<3.5cm	930 (47)	646 (53)	284 (37)	
3.5-7.2cm	695 (35)	396 (32)	299 (39)	
>7.2cm	363 (18)	188 (15)	175 (23)	
AFP, n (%)				< 0.001***
Negative	679 (34)	465 (38)	214 (28)	
Positive	1309 (66)	765 (62)	544 (72)	
Fibrosis score, n (%)				< 0.001***
F0	554 (28)	392 (32)	162 (21)	
F1	1434 (72)	838 (68)	596 (79)	
Radiotherapy, n (%)				0.002**
No	1842 (93)	1158 (94)	684 (90)	
Yes	146 (7)	72 (6)	74 (10)	
Surgery, n (%)				< 0.001***
No surgery	634 (32)	226 (18)	408 (54)	
Hepatectomy	674 (34)	569 (46)	105 (14)	
Hepatectomy and transplant	403 (20)	232 (19)	171 (23)	
Others	277 (14)	203 (17)	74 (10)	
Survival times, Median (IQR)	24 (12, 44)	27 (13, 48)	21 (11, 39.75)	< 0.001***

*, two-sided P values < 0.05.

**, two-sided P values < 0.01.

***, two-sided P values < 0.001.

AJCC, American Joint Committee on Cancer (7th).

F0, fibrosis score 0-4, non to moderate fibrosis; F1, fibrosis score 5-6, severe fibrosis and cirrhosis.

IQR, Inter-Quartile Range.
