# Supplementary material for: Association between chemotherapy and prognostic factors of survival in hepatocellular carcinoma: a SEER population-based cohort study
Source: Sci Rep. 2021 Dec 9;11:23754. doi: 10.1038/s41598-021-02698-x (PMC8660869; doi:10.1038/s41598-021-02698-x)
Supplement: Supplementary file 2 — Supplementary Information 2. [file 41598_2021_2698_MOESM2_ESM.pdf]

S2. Patients' demographics and clinicopathological characteristics grouped by AJCC stage.

| Characteristics | AJCC I (n = 426) | AJCC II (n=389) | AJCC III (n=185) | AJCC IV (n=92) | p value |
|---|---|---|---|---|---|
| Age at diagnosis, n (%) | | | | | 0.67 |
| <59 years | 196 (46) | 178 (46) | 91 (49) | 39 (42) | |
| 59-66 years | 136 (32) | 132 (34) | 50 (27) | 29 (32) | |
| 66-74 years | 94 (22) | 79 (20) | 44 (24) | 24 (26) | |
| Sex, n (%) | | | | | 0.26 |
| Female | 101 (24) | 71 (18) | 40 (22) | 17 (18) | |
| Male | 325 (76) | 318 (82) | 145 (78) | 75 (82) | |
| Race, n (%) | | | | | 0.39 |
| White | 286 (67) | 274 (70) | 117 (63) | 59 (64) | |
| Black | 64 (15) | 51 (13) | 24 (13) | 16 (17) | |
| Other | 76 (18) | 64 (16) | 44 (24) | 17 (18) | |
| Grade, n (%) | | | | | < 0.001*** |
| Well differentiated | 167 (39) | 120 (31) | 37 (20) | 22 (24) | |
| Moderately differentiated | 193 (45) | 213 (55) | 101 (55) | 44 (48) | |
| Poorly differentiated | 63 (15) | 53 (14) | 41 (22) | 26 (28) | |
| Undifferentiated | 3 (1) | 3 (1) | 6 (3) | 0 (0) | |
| Tumor size, n (%) | | | | | < 0.001*** |
| <3.5cm | 229 (54) | 252 (65) | 12 (6) | 11 (12) | |
| 3.5-7.2cm | 147 (34) | 21 (5) | 99 (54) | 50 (54) | |
| >7.2cm | 50 (12) | 116 (30) | 74 (40) | 31 (34) | |
| AFP, n (%) | | | | | 0.02* |
| Negative | 151 (35) | 112 (29) | 47 (25) | 21 (23) | |
| Positive | 275 (65) | 277 (71) | 138 (75) | 71 (77) | |
| Fibrosis score, n (%) | | | | | < 0.001*** |
| F0 | 91 (21) | 67 (17) | 61 (33) | 34 (37) | |
| F1 | 335 (79) | 322 (83) | 124 (67) | 58 (63) | |
| Treat, n (%) | | | | | < 0.001*** |
| N | 63 (15) | 35 (9) | 47 (25) | 35 (38) | |
| C | 83 (19) | 48 (12) | 43 (23) | 19 (21) | |
| R | 13 (3) | 9 (2) | 11 (6) | 6 (7) | |
| S | 130 (31) | 153 (39) | 28 (15) | 5 (5) | |
| SR | 1 (0) | 7 (2) | 3 (2) | 0 (0) | |
| SC | 130 (31) | 124 (32) | 37 (20) | 13 (14) | |
| RC | 6 (1) | 7 (2) | 10 (5) | 9 (10) | |
| SRC | 0 (0) | 6 (2) | 6 (3) | 5 (5) | |
| Survival times, Median (IQR) | 28.00 (15.00, 53.00) | 29.00 (16.00, 50.00) | 11.00 (4.00, 25.00) | 6.50 (2.00, 14.00) | < 0.001*** |

*, two-sided P values < 0.05.

AJCC, American Joint Committee on Cancer (7$^{th}$).

Fibrosis score, F0, fibrosis score 0-4, non to moderate fibrosis; F1, fibrosis score 5-6, severe fibrosis and cirrhosis.

Treat, N, no treatment; C, chemotherapy alone; R, radiotherapy alone; S, surgery alone; SR, surgery combined with radiotherapy; SC, surgery combined with chemotherapy; RC, radiotherapy combined with chemotherapy; SRC, surgery combined with radiotherapy and chemotherapy.
