# Supplementary material for: Association between chemotherapy and prognostic factors of survival in hepatocellular carcinoma: a SEER population-based cohort study
Source: Sci Rep. 2021 Dec 9;11:23754. doi: 10.1038/s41598-021-02698-x (PMC8660869; doi:10.1038/s41598-021-02698-x)
Supplement: Supplementary file 3 — Supplementary Information 3. [file 41598_2021_2698_MOESM3_ESM.pdf]

S3. Competing risk analysis of cancer-specific death in HCC patients grouped by AJCC stage.

Characteristics	AJCC I (n=131）			AJCC II (n=138）			AJCC III (n=122)			AJCC IV (n=76)		
	N	HR (95% CI)	P value	N	HR (95% CI)	P value	N	HR (95% CI)	P value	N	HR (95% CI)	P value
Age at diagnosis, n (%)												
<59 years	62	Reference		52	Reference		58	Reference		32	Reference	
59-66 years	37	0.96(0.60-1.54)	0.86	53	1.57(1.05-2.33)	0.03*	32	1.12(0.69-1.81)	0.65	26	1.43(0.84-2.41)	0.19
66-74 years	32	1.05(0.66-1.67)	0.84	33	1.68(1.08-2.59)	0.02*	32	1.14(0.75-1.75)	0.54	18	1.40(0.76-2.56)	0.28
Sex, n (%)												
Female	26	Reference		28	Reference		30	Reference		14	Reference	
Male	105	1.17(0.74-1.86)	0.5	110	0.73(0.45-1.16)	0.18	92	0.59(0.40-0.85)	0.005**	62	0.63(0.36-1.13)	0.12
Race, n (%)												
White	92	Reference		98	Reference		80	Reference		48	Reference	
Black	22	1.01(0.57-1.79)	0.98	18	0.66(0.39-1.11)	0.12	13	0.91(0.44-1.90)	0.8	14	1.71(1.02-2.89)	0.04*
Other	17	0.66(0.37-1.18)	0.16	22	0.8(0.47-1.35)	0.4	29	0.88(0.58-1.32)	0.53	14	2.15(1.20-3.85)	0.01*
Grade, n (%)												
Well differentiated	51	Reference		41	Reference		21	Reference		17	Reference	
Moderately differentiated	50	0.99(0.65-1.50)	0.96	72	1.08(0.72-1.64)	0.7	65	1.61(0.95-2.73)	0.08	34	1.34(0.72-2.49)	0.36
Poorly differentiated	29	2.31(1.34-3.99)	0.003**	25	1.51(0.94-2.43)	0.09	31	3.06(1.67-5.58)	<0.001***	25	2.02(0.99-4.12)	0.053
Undifferentiated	1	1.32(0.08-21.90)	0.85	0	0.00001(0.000002-0.00005)	<0.001***	5	2.53(1.14-5.59)	0.02*	0	—	—
Tumor size, n (%)												
<3.5cm	43	Reference		68	Reference		8	Reference		10	Reference	
3.5-7.2cm	59	1.84(1.19-2.85)	0.006**	61	2.71(1.90-3.88)	<0.001***	45	0.77(0.36-1.66)	0.51	24	0.66(0.30-1.43)	0.29
>7.2cm	29	3.67(2.18-6.19)	<0.001***	9	2.2(0.89-5.44)	0.09	69	0.94(0.45-1.94)	0.86	42	1.00(0.48-2.12)	0.99
AFP, n (%)												
Negative	45	Reference		33	Reference		29	Reference		14	Reference	
Positive	86	1.23(0.79-1.92)	0.37	105	1.2(0.79-1.84)	0.39	93	1.23(0.82-1.84)	0.33	62	1.94(1.06-3.55)	0.03*
Fibrosis score, n (%)												
F0	27	Reference		25	Reference		37	Reference		27	Reference	
F1	104	0.83(0.51-1.33)	0.44	113	0.86(0.52-1.42)	0.55	85	1.27(0.85-1.90)	0.25	49	0.97(0.57-1.67)	0.92
Treat, n (%)												
N	37	Reference		24	Reference		39	Reference		34	Reference	
C	46	0.7(0.42-1.17)	0.18	25	0.45(0.24-0.84)	0.01*	33	0.50(0.29-0.87)	0.01*	15	0.47(0.24-0.91)	0.03*
S	13	0.1(0.05-0.21)	<0.001***	39	0.15(0.09-0.26)	<0.001***	10	0.15(0.07-0.30)	<0.001***	5	0.44(0.23-0.84)	0.01*
SC	26	0.21(0.12-0.38)	<0.001***	39	0.21(0.13-0.36)	<0.001***	18	0.19(0.10-0.33)	<0.001***	7	0.14(0.05-0.36)	<0.001***
Others	9	0.62(0.29-1.33)	0.22	11	0.31(0.16-0.57)	<0.001***	22	0.48(0.26-0.86)	0.01*	15	0.32(0.19-0.55)	<0.001***

*, two-sided P values < 0.05.

AJCC, American Joint Committee on Cancer (7th).

Fibrosis score, F0, fibrosis score 0-4, non to moderate fibrosis; F1, fibrosis score 5-6, severe fibrosis and cirrhosis.

Treat, N, no treatment; C, chemotherapy alone; R, radiotherapy alone; S, surgery alone; SR, surgery combined with radiotherapy; SC, surgery combined with chemotherapy; RC, radiotherapy combined with chemotherapy; SRC, surgery combined with radiotherapy and chemotherapy.
